# Acknowledgements are not just thank you notes: A qualitative analysis of acknowledgements content in scientific articles and reviews published in 2015

**DOI:** 10.1371/journal.pone.0226727

**Published:** 2019-12-19

**Authors:** Adèle Paul-Hus, Nadine Desrochers

**Affiliations:** École de bibliothéconomie et des sciences de l'information, Université de Montréal, Downtown Station, Montreal, Quebec, Canada; Indiana University Bloomington, UNITED STATES

## Abstract

Acknowledgements in scientific articles can be described as miscellaneous, their content ranging from pre-formulated financial disclosure statements to personal testimonies of gratitude. To improve understanding of the context and various uses of expressions found in acknowledgements, this study analyses their content qualitatively. The most frequent noun phrases from a Web of Science acknowledgements corpus were analysed to generate 13 categories. When 3,754 acknowledgement sentences were manually coded into the categories, three distinct axes emerged: the contributions, the disclaimers, and the authorial voice. Acknowledgements constitute a space where authors can detail the division of labour within collaborators of a research project. Results also show the importance of disclaimers as part of the current scholarly communication apparatus, an aspect which was not highlighted by previous analyses and typologies of acknowledgements. Alongside formal disclaimers and acknowledgements of various contributions, there seems to remain a need for a more personal space where the authors can speak for themselves, in their own name, on matters they judge worth mentioning.

## Introduction

The idea of using acknowledgements as a source for bibliometric indicators has been surrounding their study since the 1990s. In 1991, Cronin was already asking, “why are acknowledgement counts excluded from formal assessments of individual merit or influence, such as tenure review?” ([1]: p. 236). In 1995, Cronin and Weaver were encouraging the development of an Acknowledgement Index, based on the model of the Science Citation Index [2]. Almost two decades later, Costas and van Leeuwen [3] suggested that it was perhaps time “to employ this sort of tool to facilitate development of the so-called ‘influmetrics’” ([3]: p. 1659). For their part, Díaz-Faes and Bordons [4] highlighted that the inclusion of acknowledgement information in the Web of Science (WoS) was offering new avenues to study collaboration in science, going beyond traditional bibliometric indicators. McCain [5] went further and assessed the feasibility of a formal Personal Acknowledgements Index. And yet, despite decades of studies positioning acknowledgements alongside citations and authorship in what Cronin called the “reward triangle” [6], the consideration of acknowledgements as an indicator of scientific credit has not materialized and, at best, remains a proposal at the exploratory stage, or even simply a rhetorical idea (see [7] for a meta-synthesis of this literature).

At the same time, many studies have used funding-related indicators based on acknowledgement data (e.g. [8–11]). In fact, acknowledgement studies can no longer be separated from the financial aspect of scientific research. In 2008, WoS started to collect and index funding sources found in the acknowledgements of scientific papers. These new data were added by WoS in response to many funding bodies’ requirement to acknowledge the sources supporting research. Since then, large-scale acknowledgement data have been used as a bibliometric tool to follow the money trail of research and funding-related analyses have become a dominant trend in recent acknowledgement literature [7]. To this day, acknowledgements have been more closely related to funding indicators than to any other kind of scientific credit indicators.

The literature also underlines the elusive nature of acknowledgements, pointing to their form and tone, which have been described as sometimes flowery, personal, and even manipulative:

Acknowledgements are permeated by hyperbole, effusiveness, overstatement, and exaggeration. ([12]: p. 64)
Acknowledgements have been discussed as a form of patronage in scholarly communication, where the reality of the past may be purposefully glossed over and where the author could be looking toward the possibility of receiving future favours. ([13]: p. 4)

Furthermore, several studies mention the lack of standardization of acknowledgements as one important limitation hindering their analyses:

The format of acknowledgement varies from field to field and from journal to journal. As noted, persons and institutional sources may be listed in the methods and materials section of an article or explicitly thanked in an acknowledgement section. ([14]: p. 506)
Since there are no established formats for acknowledgements in papers, as there are for citations, expressions of gratitude vary greatly and sometimes it was difficult to identify the correct type of support, and even more difficult, the correct funding organization. ([15]: p. 238)
The first source of simple error may arise through the misspelling of the names of funding bodies and potentially the names of grants and grant codes […]. A second difficulty will be that researchers will not correctly remember the funding bodies and grants that they used to support the research. ([16]: p. 368–369)

Acknowledgements may thus contain formally required statements of gratitude but have also been used as personal spaces of authorial expression, and as such, acknowledgement texts have been analysed as a genre per se. Several discourse and linguistic analyses have studied acknowledgements found in dissertations, theses, monographies, and research articles (e.g. [17–19]).

Acknowledgements analyses have also led to numerous typologies or classifications of the contributions acknowledged in scientific publications. In 1972, Mackintosh [20] proposed the first qualitative content analysis of acknowledgements based on a typology of the three main types of “services” acknowledged in scientific papers: *facilities*, *access to data*, and *help of individuals*. Twenty years later, McCain [14] offered a finer typology of acknowledgements, using five categories: *access to research-related information*, *access to unpublished results and data*, *peer interactive communication*, *technical assistance*, and *manuscript preparation*. The same year, Cronin introduced his first version of a six-part typology of acknowledgements (*paymaster*, *moral support*, *dogsbody*, *technical*, *prime mover*, and *trusted assessor*) which was created before encountering Mackintosh’s 1972 and McCain’s 1991 work [1,21]. Subsequent versions of this typology—developed with different collaborators through the years (namely McKenzie, Rubio and Weaver(-Wozniak))—include the *peer interactive communication* category borrowed from McCain [14] alongside *moral support*, *access* (to resources, materials and infrastructure), *clerical support*, *technical support*, and *financial support* [2,22–24]. Cronin’s model has since been adopted, adapted, and augmented in several studies (e.g. [25–30].

More recently, Giles and Councill [31] used natural language processing to extract named entities from more than 180,000 acknowledgements published in computer science research papers. In their content analysis, the most frequently acknowledged entities are classified into four categories: *funding agencies*, *corporations*, *universities* and *individuals*. Other studies have analysed the content of acknowledgements focusing on funding bodies and classifying them by sectors and subsectors (e.g. [10,32–35]).

Finally, linguistic studies have also used classifications of acknowledgements, focusing on the structure and patterns of dissertation acknowledgement texts (e.g. [18,36–40]) and on the socio-pragmatic construction of acknowledgements found in research articles and academic books [19,41–43].

Typologies and classifications aim to describe and categorize the content of acknowledgements in a synthetic manner. However, these taxonomies are based on small-scale samples of acknowledgements, the only exception being the work of Giles and Councill [31] which focused solely on named entities. More recently, a large-scale multidisciplinary analysis of acknowledgement texts was published by the authors and collaborators in PLOS One [44]. This analysis of acknowledgements from more than one million articles and reviews published in 2015, highlighted important variations in the practices of acknowledging. Focusing on the 214 most frequent noun phrases of that corpus, the study showed that acknowledgement practices truly do vary across disciplines. Noun phrases referring to technical support appeared more frequently in natural sciences while noun phrases related to peers (colleagues, editors and reviewers) were more frequent in earth and space, professional fields, and social sciences. Noun phrases referring to logistics and fieldwork-related tasks appeared prominently in biology. Pre-formulated statements used in the context of conflict of interest or responsibility disclosures were more frequently found in acknowledgements from clinical medicine, health, and psychology. However, this analysis also led to further questions concerning the interpretation of these noun phrases in their original context. Findings from this study showed that acknowledgements are not limited to credit attribution and that the numerous taxonomies and classifications found in the literature do not account for the current acknowledgement practices where pre-formulated statements of financial assistance and conflict of interest disclosures appear to be frequent [44]. Conclusions from this study raise further questions because these pre-formulated statements could have an influence on large-scale analyses that use automated linguistic methods, thus calling for a qualitative analysis of acknowledgements in the context of their use.

### Objective and research questions

To improve understanding of the context and various uses of expressions found in acknowledgements, this study proposes to analyse their content qualitatively. More specifically, this study aims at answering the following research questions:

- In which contexts are specific expressions used?
- Do the contexts and meanings vary by discipline?
- What does a qualitative analysis reveal in terms of offering avenues for a more contextualized use of acknowledgements in large-scale studies?

## Data and methods

Data for this study were retrieved from WoS’s Science Citation Index Expanded (SCI-E) and Social Sciences Citation Index (SSCI), which both include funding acknowledgement data. It bears repeating that acknowledgments are collected and indexed by WoS only if they include funding source information [45]. Access to WoS data in a relational database format was provided by the *Observatoire des sciences et des technologies* (http://www.ost.uqam.ca). The full text of acknowledgements from all 2015 articles and reviews indexed in the SCI-E and the SSCI were extracted. The original corpus includes a total of 1,009,411 acknowledgements for as many papers.

In a previous analysis, we identified the 214 most frequent noun phrases of that corpus of acknowledgement using natural language processing [44]. For the purpose the present qualitative analysis, these 214 noun phrases were reduced to single words (e.g. “technical assistance” was reduced to “technical” and “assistance”) and redundant words were excluded, for a final corpus of 154 single words. Each single word could therefore be found in context, no matter its proximity to other single words; this offered us the possibility to code various types of occurrences of each word, whether it was part of a noun phrase or not.

The coding was done in two steps. First, an initial codebook was established inductively by one researcher to classify each of the 154 words and revised by a second researcher. All words were then coded by both researchers and their work was reconciled through “negotiated agreement” ([46]: p. 305, see also [47,48]). Second, 20 words were selected from the corpus of 154 words by purposeful sampling, where cases for study are selected because “they offer useful manifestations of the phenomenon of interest” ([49]: p. 40). Selection of the words included in the final sample was based on the quantitative analysis findings [44], which highlighted the potential importance of pre-formulated statements such as “The funders had no role in study design, data collection and analysis, decision to publish, or preparation of the manuscript” (ut 000367510900041). Special attention was given to the words frequently used in those statements (e.g. analysis, collection, design, preparation). Sampling decisions were also oriented towards potential polysemous words which could lead to different contextual meanings (e.g. “assistance”). The 20 words of the final sample were coded within the context of their original sentences, extracted from acknowledgements. Words were thus used as a seed to refer back to full acknowledgement sentences.

The coding process entails data reduction where the many meanings of a sentence must be reduced or summarized under one main category [50] in order to reflect a practice or a phenomenon on a humanly manageable scale. The principles of saturation and qualitative sampling, whereby the sample is “conceptually representative of the set of all possible units” ([51]: p. 84), ensures that the phenomenon is reflected in its full complexity. Therefore, acknowledgements were stratified by discipline to reflect potentially different disciplinary uses of a word. Coding was then performed on this sample of 20 words within their original acknowledgement contexts, using the sentence as the unit of analysis and adapting the codebook in an iterative manner as finer meanings emerged.

The final codebook is composed of 13 categories, presented in Table 1. The coding was done by one researcher and guided by the question, “in which context is this word used?” One category was selected for each sentence coded, aiming at qualifying the context in which a word is used. Each word of the sample was coded in a minimum of 15 original sentences per discipline, for all 12 disciplines, resulting in a total of 3,754 sentences coded. Results are reported in “thick description” using sufficient descriptions and quotations to allow “thick interpretation”, which means connecting individual cases to the larger context without going into trivial details ([49]: p. 503).

**Table 1:** Codebook: Categories of acknowledgement content and their definitions.

Category	Definition	Example
Financial disclosure	Includes all types of funding and financial support or assistance.	“The financial assistance of the National Research Foundation (NRF grant: Unlocking the future- FA2007043000003) towards this research is hereby acknowledged.” (ut 000350024900008)
Conflict of interest	Refers to potential or actual conflict of interest or the absence of conflict of interest, which can be financial or otherwise.	“P.A.P. has an equity interest in Digital Proteomics, LLC, a company that may potentially benefit from the research results.” (ut 000356625700007)
Disclaimer	Responsibility disclaimer that content/opinions/conclusions are those of the author(s) solely and not of the funder or of another organization.	“The funders had no role in study design, data collection and analysis, decision to publish, or preparation of the manuscript.” (ut 000366223600042)
Ethics	Refers to ethical review, ethical approval of the research; can include some form of "seal of approval" by agencies.	“Institutional review board approval was granted under University IRB PRO12110345.” (ut 000364165000006)
Peer communication	Refers to intellectual contribution and communication with colleagues and peers, trusted assessors. Includes the process of comments, feedback, suggestions and peer review.	“Tim Birt, David Anderson, Anna Tigano, Rebecca Taylor, Nathaniel Clark, Catherine Dale and Raphael Lavoie provided insightful discussions.” (ut 000367457200004)
Investigation and Analysis	Refers to specific tasks such as the collection, treatment and analysis of data; the cycle of pre-writing work.	“Thanks Dr. Dongliang Li and Dr. Jianjun Cao from Nanjing Xiaozhuang University, for their help on field work and data analysis.” (ut 000350479600001)
Supervision and Management	Tasks and roles related to supervision, leadership and management responsibilities.	“Research included in this review was partly completed at the University of Newcastle, Australia, under the supervision of Dr John Clulow and Dr Micheal Mahony.” (ut 000346218400001)
Materials and Resources	Refers to all kinds of study materials, samples, computing resources, infrastructure, physical installations and instrumentation and reagents. People as objects of study (such as patients or population/sample) are also included.	“We thank Calcul Quebec and Compute Canada for access to the Mammoth supercomputer.” (ut 000363365000021)
Writing	Includes creation and/or presentation of the published work: original draft preparation, contribution to the writing itself; can include the creation of visualizations, maps, figures, tables, and illustrations.	“We thank Donald Cochrane, University of Saskatchewan, for his writing assistance” (ut 000353426000019)
Dissemination	Includes project, documents, and other forms of dissemination, such as conference presentations. Includes issues linked to cost of publication and open-access models.	“Data and supporting materials necessary to reproduce the numerical results will be available at www.hobolt.com upon publication.” (ut 000350337600013)
Organization	Refers to institutions or organizations, research centres, research groups, research chairs (can include funding organizations).	“The second author would like to thank Guangxi Experiment Center of Information Science.” (ut 000353065700007)
Combination	Two or more clear categories combined.	“The authors are grateful to the two referees and the editor for comments and suggestions and to Alfio Viola (University of Catania) for SEM assistance.” (ut 000353204900002)
Vague or other	Meaning cannot be inferred or is not covered by any other categories.	“We thank Jonas Klevas and Dainius Prakapavicius for their contribution during various stages of the paper preparation.” (ut 000357274600063)

## Results

The results of the coding process are summarized in Table 2 which presents, for each word of the sample, the percentage of all the occurrences attributed to a specific category. The analysis reveals the importance of three distinct axes: the contributions, the disclaimers, and the authorial voice. Moreover, disciplinary patterns bring another layer of analysis as divergent uses of the coded words emerge.

**Table 2:** Acknowledgement words coding results.

	Investigation & analysis	Financial disclosure	Disclaimer	Peer comm.	Materials & resources	Dissemin.	Writing	Conflict of interest	Org.	Ethics	Other *
work	1%	89%	0%	1%	3%	1%	1%	1%	1%	0%	2%
author	10%	41%	9%	16%	8%	0%	1%	10%	1%	0%	6%
analysis	47%	8%	32%	7%	6%	0%	0%	0%	0%	0%	0%
preparation	21%	17%	35%	12%	4%	0%	7%	0%	0%	0%	4%
assistance	62%	19%	0%	1%	2%	0%	7%	0%	0%	0%	8%
help	65%	2%	0%	4%	1%	0%	10%	0%	0%	0%	18%
data	29%	3%	24%	1%	40%	3%	0%	0%	1%	0%	0%
decision	2%	28%	65%	0%	0%	1%	0%	0%	4%	0%	1%
contribution	28%	18%	0%	7%	0%	8%	1%	0%	1%	0%	37%
discussion	0%	1%	0%	98%	1%	1%	0%	0%	0%	0%	0%
experiment	62%	5%	2%	5%	15%	1%	0%	0%	9%	1%	0%
results	1%	52%	15%	14%	5%	9%	0%	1%	0%	1%	2%
access	0%	2%	3%	0%	70%	23%	0%	1%	0%	1%	0%
review	2%	1%	7%	58%	0%	25%	6%	0%	0%	2%	0%
collection	54%	5%	38%	0%	3%	0%	0%	0%	0%	0%	0%
measurement	64%	13%	1%	2%	14%	3%	0%	1%	2%	1%	1%
writing	5%	20%	36%	18%	4%	0%	16%	0%	0%	0%	1%
design	22%	2%	55%	10%	1%	2%	2%	0%	6%	0%	1%
interpretation	25%	1%	61%	13%	0%	0%	0%	0%	0%	0%	0%
code	45%	53%	0%	0%	0%	2%	0%	0%	0%	1%	0%
Total	26%	22%	18%	13%	9%	4%	2%	1%	1%	0%	4%

Words are presented in the table in descending order of their frequency in the corpus.

* “Other” regroups the following categories: Supervision and Management, Combination, and Vague or other.

### Contributions

Acknowledgements constitute a space where authors can detail “who has done what” during the research process. Most often, authors use this space to thank colleagues that contributed to the research, as in the following example: “The authors thank Colleen Dalton and four anonymous reviewers for their helpful comments that improved the manuscript. We thank Fan-Chi Lin for providing FTAN measurements for comparison, and Anna Foster, Jiayi Xie and Goran Ekstrom for informative discussion.” (ut 000355321800013; earth and space). However, in some cases acknowledgements can also include contributorship statements from the authors in order to reflect the distribution of labour: “A.P., V.M. and V.P were involved in writing the manuscript. A.B.G and Y.A.K. were responsible for conception of the idea” (ut 000365808000014; clinical medicine).

The categories peer communication, investigation and analysis, materials and resources, and writing refer to specific types of contribution to research. These categories, taken together, represent half (50%) of the sample coded, confirming the importance of the contributions axis within the acknowledgements’ context. Moreover, some words are used most often to refer to specific categories of contribution, such as “access” which is used mainly in the category materials and resources (70% of the occurrences coded), “discussion” which is almost exclusively associated to the peer communication category (98% of the occurrences coded), and “assistance”, “experiment”, “help”, and “measurement”, which are all mainly associated to the category investigation and analysis (more than 60% of the occurrences coded).

### Disclaimers

Acknowledgements are not necessarily thank-you notes or recognition of responsibility. Financial disclosure, conflict of interest, disclaimer, and ethics account for more than 40% of the sample coded. In fact, the categories financial disclosure and disclaimer are among the most frequent in the sample, accounting respectively for 22% and 18% of all occurrences coded. The words “analysis”, “collection”, “decision”, “design”, “interpretation”, “preparation”, and “writing”, which could all seemingly refer to types of contributions, were in fact used in the context of responsibility statements in a substantial share of the cases analysed. Moreover, the words “decision”, “design” and “interpretation” also are mostly found in those kinds of responsibility disclaimers (in respectively 65%, 55% and 61% of the occurrences coded for these specific words).

Non-responsibility statements of funding bodies are the most frequent disclaimers. The following example presents a typical statement: “The funding source had no role in the design of the study, the analysis and interpretation of the data or the writing of, nor the decision to publish the manuscript.” (ut 000352854700010). However, we found declarations of non-responsibility for other types of contributors regarding some part of a research project, as in the following sentence: “The data collectors have no responsibility over the analysis and interpretations presented in this study.” (ut 000349266800011). Furthermore, disclaimers are not always non-responsibility statements and can, on the contrary, disclose the specific responsibility of an organization, such as: “This study was funded by Xi'an Janssen Pharmaceutical Ltd (Beijing, People's Republic of China) who was responsible for study design and data collection, analysis, and interpretation.” (ut 000356594900001).

### Contributions and disclaimers crossovers

In many cases, the disciplinary stratification provided a further level of analysis. The words “analysis”, “assistance”, and “code” present clear disciplinary patterns where the coding highlights the distinction between the two main contextual uses: the contributions axis and the disclaimers axis. For instance, the word “analysis” is used primarily in the sample to describe an investigation and analysis type of contribution: “We are grateful to Nahoko Adachi for her help in conducting the statistical analysis” (ut 000353959400005; psychology). However, for biomedical research, clinical medicine, and health, “analysis” is used mainly within the category disclaimer (example: “The funding agencies did not have any role in study design, data collection and analysis, decision to publish, or preparation of the manuscript” [ut 000346498800018; clinical medicine]). Mathematics is a divergent discipline, where the dominant category for “analysis” is financial disclosure, as exemplified by the following sentence: “This work was supported by the International Max-Planck Research School, 'Analysis, Design and Optimization in Chemical and Bio-chemical Process Engineering', Otto-von-Guericke-Universitat Magdeburg” (ut 000362588800005; mathematics).

Similarly, the word “assistance” is generally used across disciplines to describe a contribution pertaining to the category investigation and analysis (example: “The authors thank S. Watmough and K. Finder for assistance with field sampling at Dorset, and A. McDonough for assistance with the classification of plant species” [ut 000347756900044; earth and space]), except in engineering and technology and in mathematics where “assistance” is used to disclose financial help (financial disclosure) in the majority of the cases examined, as in this sentence: “The financial assistance of the National Research Foundation (NRF grant: Unlocking the future- FA2007043000003) towards this research is hereby acknowledged” (ut 000350024900008; mathematics).

Two distinct contextual uses emerge for the word “code”: it is found most often within the disclaimers axis (financial disclosure category) in biology, biomedical research, chemistry, health, psychology and social sciences (example: “The research (project code: TSY-11-3820) was supported by the Research Fund of Erciyes University” [ut 000363704000011; biology]) while it is used to describe a specific contribution (investigation and analysis category) in the majority of the cases studied in earth and space, engineering and technology, mathematics, physics and professional fields (example: “We thank Prof. D. Karaboga and Dr. B. Basturk for providing their excellent ABC MATLAB codes to implement this research” [ut 000361400900022; earth and space]).

In the case of the word “review”, the coding process also highlights two dominant uses, varying with the discipline: in biology, biomedical research, earth and space, mathematics, physics, and in the professional fields, “review” is used primarily to describe some part of the peer communication process (peer communication category), as in the following example: “We would like to express our gratitude to the anonymous referee for his or her careful review and insightful comments, in particular, for pointing out a simple proof of Lemma 1.8.” (ut 000347714700003; engineering and technology). However, in clinical medicine, a different use is made of the word “review,” mainly to refer to the document per se (dissemination category), as in this example: “We are grateful to Dr. Mozzetta for critically reading the manuscript and all members of the lab for stimulating discussions during the preparation of this review” (ut 000352374400001; clinical medicine). For all the remaining disciplines (chemistry, health, psychology, and social sciences), both categories (peer communication and dissemination) appear frequently.

The word “data” also presents distinct disciplinary patterns in the sample coded. “Data” is used mainly within the contributions axis (materials and resources category) in biology, clinical medicine, earth and space, engineering and technology, and social sciences (example: “The authors thank Chesapeake Energy for providing access to the VSP data we used” [ut 000364362900035; earth and space]). Moreover, the word “data” refers to a task within the investigation and analysis category in an important share of the cases coded in chemistry, physics, professional fields, and psychology (example: “We thank all graduate research assistants who helped with data collection” [ut 000348882900009; psychology]). However, “data” is mainly found within the disclaimers axis in clinical medicine and health (disclaimer category) as in the following example: “The funding agencies had no role in the study design, data collection and analysis, the decision to publish or preparation of the manuscript” [ut 000345586900003; clinical medicine].

### Authorial voice

Although details of contributions and various disclaimers represent a substantive share of their content, acknowledgements also constitute a space for personal testimony. Notwithstanding the expectations of funders and ethical considerations, acknowledgements remain the subjective presentation of researchers’ practices and of research contexts. The authors are the voice of the acknowledgements and as such, the word “author” is one of the most frequent with more than 339,000 occurrences in our dataset. Moreover, even when the word “author” is absent, the concept is not. In fact, the authorial voice cannot be reduced to a single category, because it pervades the acknowledgements whether the authors speak in the first or third persons:

“**I** would like to thank Iliana Flores, Amy Harrison, and Shannon Kahlden for their help with data collection.” (ut 000361977300090)
“**We** would also like to thank two anonymous reviewers for the contributions to this manuscript.” (ut 000364777400031)
“Also, **our** thanks go to Mr Vit Hanousek who designed an original computer tool suitable for making all the above-discussed measurements.” (ut 000346267600010)
“The **authors** declare that they have no competing interests.” (ut 000369908800022)
“The **authors** wish to express their appreciation to the National Iranian Copper Industry Company (NICICO) for funding this work.” (ut 000344595900005)
“Schuster is profoundly grateful to all the families who hosted **her** but especially Hasidullah, his wife, son and grandson who were unfailingly patient and kind with the strange cuckoo in their nest and to the Leverhulme Trust for funding **her** time in Afghanistan.” (ut 000350285300006)
“This review is dedicated to the memory of **my** father who was a source of inspiration.” (ut 000349637500005)

Furthermore, as exemplified by the cases presented above, the varied nature of the testimonies found in acknowledgements underlines a need for a “free space” within research publications. Alongside formal disclaimers and acknowledgements of various contributions, authors seem to require a more personal space where they can speak for themselves, in their own name, on matters they judge worth mentioning.

## Discussion and conclusion

In the last decades, acknowledgements have become a “constitutive element of academic writing” ([52]: p. 160). However, the acknowledgement section is not a mandatory part of a scientific article and its content could certainly be described as miscellaneous, ranging from pre-formulated financial disclosure statements to personal testimonies of gratitude. Moreover, acknowledgements’ content and practices have evolved over time, just as citations and authorship attribution practices have changed following the transformations that are affecting the whole reward system of science [53].

Typologies and classifications of acknowledgements have been a consistent topic in the acknowledgement literature [7]. Most of these typologies and classifications revolve around the contributions axis of acknowledgements, focusing on “who gets thanked for what” and “what types of contributions are acknowledged”. This qualitative analysis of acknowledgement content confirms the importance of the contributions axis: acknowledgements are indeed still a space where authors can detail the division of labour within all collaborators of a research project. Our findings also reveal the importance of disclaimers as part of the current scholarly communication apparatus, an aspect which was not highlighted by previous analyses and typologies.

It should be noted that our analysis was restricted to a corpus of single words, sampled from noun phrases identified by correspondence analysis [44]. Further research could now seek to recombine those single words into noun phrases that present variations in meaning around a common concept, such as “assistance” (e.g. “technical assistance” and “financial assistance”). Furthermore, our coding of acknowledgement sentences was done using mutually exclusive categories, an epistemological choice. Given the fact that sentences can perform more than one kind of action, another avenue would be to use open coding and place occurrences in non-exclusive, mutually complementary categories.

Our qualitative results show that caution should be used when working with acknowledgement data. Large-scale acknowledgement data are limited to funded research, given that in the two main bibliographic databases, Web of Science and Scopus, acknowledgements are collected with the intended objective of identifying funding sponsors and tracking funded research [54,55]. The indexation of acknowledgements are thus limited to acknowledgements that contain some kind of funding information; this could in turn induce a potential bias toward funding-related aspects within acknowledgements’ content [45]. This indexation bias could then, at least in part, explain the importance of funding disclosures in the dataset analysed here, but also elsewhere in large-scale studies.

Yet, our findings show that acknowledgements cannot be described as having one single and homogeneous purpose; they can include expected, if not imposed, acknowledgement of financial resources as well as infrastructure alongside very personal testimonies of gratitude, all at the same time, as the following excerpt exemplifies: “Data presented herein were obtained at the W. M. Keck Observatory, which is operated as a scientific partnership among the California Institute of Technology, the University of California, and the National Aeronautics and Space Administration. […]. The authors wish to extend special thanks to those of Hawaiian ancestry, on whose sacred mountain we are privileged to be guests. Without their generous hospitality, the observations would not have been possible” (ut 000363471600015). On rare occasions, personal matters discussed in the acknowledgements become the center of attention, such as when an author proposed to his girlfriend in the acknowledgement of a paper: “C.M.B. would specifically like to highlight the ongoing and unwavering support of Lorna O’Brien. Lorna, will you marry me?” [56]. This particular paper was covered by many news outlets and online media sites when it was published, ranking in the 20^th^ position of the Altmetrics Top100 ranking for the year 2015. Such a case highlights the potential unexpected effect an acknowledgement can have on the visibility of a paper.

Clearly delimited and dedicated spaces for funding information, conflict of interest disclosures and contributorship statements are already implemented in some scientific journals (e.g. *PLOS One*, *The Lancet*, *Science*). Nonetheless, such examples are far from the norm at the moment. In light of our findings, if an effort of standardization of acknowledgements is to be made, acknowledgements should at least include three main sections: ethics of research (financial disclosure, conflict of interest and responsibility disclaimers), contributions made to research, and personal testimony. These three indexation fields would, in turn, allow large-scale analysis of acknowledgements without the equivocality that currently characterizes these texts, yet without narrowing the space left for the authorial voice. The question remains as to whether there is a real wish within the scientific community to delineate such acknowledgement sections; if not, acknowledgement data are likely destined to remain simple tracking devices for science funding, the contributions and the authorial voices lost in large-scale analyses of scientific credit.

## Supporting information

S1 Table References of the acknowledgement excerpts cited. References are presented in order of in-text appearance.
(DOCX)
