# Supplementary material for: Acknowledgements are not just thank you notes: A qualitative analysis of acknowledgements content in scientific articles and reviews published in 2015
Source: PLoS One. 2019 Dec 19;14(12):e0226727. doi: 10.1371/journal.pone.0226727 (PMC6922370; doi:10.1371/journal.pone.0226727)
Supplement: S1 Table — References are presented in order of in-text appearance. (DOCX) [file pone.0226727.s001.docx]

S1 Table. References of the acknowledgement excerpts cited

References are presented in order of in-text appearance.

| **UT codes** | **References** |
| --- | --- |
| 000367510900041 | Arciero, E., Biagini, S. A., Chen, Y., Xue, Y., Luiselli, D., Tyler-Smith, C., … Ayub, Q. (2015). Genes Regulated by Vitamin D in Bone Cells Are Positively Selected in East Asians. *PLOS ONE*, 10(12), e0146072. <https://doi.org/10.1371/journal.pone.0146072> |
| 000350024900008 | Arashi, M., Bekker, A., Loots, M. T., & Roux, J. J. J. (2015). Integral Representation of Quaternion Elliptical Density and its Applications. *Communications in Statistics-Theory and Methods*, 44(4), 778‑789. <https://doi.org/10.1080/03610926.2012.753089> |
| 000356625700007 | Safonova, Y., Bonissone, S., Kurpilyansky, E., Starostina, E., Lapidus, A., Stinson, J., … Pevzner, P. A. (2015). IgRepertoireConstructor: a novel algorithm for antibody repertoire construction and immunoproteogenomics analysis. *Bioinformatics*, 31(12), 53‑61. <https://doi.org/10.1093/bioinformatics/btv238> |
| 000366223600042 | Pyun, S. Y., Jeong, J.-H., & Bae, J. S. (2015). Recurrent Guillain-Barre syndrome presenting stereotypic manifestations, positive antiganglioside antibodies, and rapid recovery. *Clinical Neurology and Neurosurgery*, 139, 230‑233. <https://doi.org/10.1016/j.clineuro.2015.10.022> |
| 000364165000006 | Lamberton, C. M., Leana, C. R., & Williams, J. M. (2015). Measuring Empathetic Care: Development and Validation of a Self-Report Scale. *Journal of Applied Gerontology*, 34(8), 1028‑1053. https://doi.org/10.1177/0733464813507131 |
| 000367457200004 | Friesen, V. L. (2015). Speciation in seabirds: why are there so many species...and why aren’t there more? *Journal of Ornithology*, 156, S27‑S39. https://doi.org/10.1007/s10336-015-1235-0 |
| 000350479600001 | Gu, Z., Sanchez-Azofeifa, G. A., Feng, J., & Cao, S. (2015). Predictability of leaf area index using vegetation indices from multiangular CHRIS/PROBA data over eastern China. *Journal of Applied Remote Sensing*, 9, 096085. https://doi.org/10.1117/1.JRS.9.096085 |
| 000346218400001 | Browne, R. K., Kaurova, S. A., Uteshev, V. K., Shishova, N. V., McGinnity, D., Figiel, C. R., … Cosson, J. (2015). Sperm motility of externally fertilizing fish and amphibians. *Theriogenology*, 83(1), 1‑13. https://doi.org/10.1016/j.theriogenology.2014.09.018 |
| 000363365000021 | Vapaavuori, J., Grosrenaud, J., Pellerin, C., & Bazuin, C. G. (2015). In Situ Photocontrol of Block Copolymer Morphology During Dip-Coating of Thin Films. *Acs Macro Letters*, 4(10), 1158‑1162. https://doi.org/10.1021/acsmacrolett.5b00483 |
| 000353426000019 | Bisol, C. A., Valentini, C. B., & Rech Braun, K. C. (2015). Teacher education for inclusion: Can a virtual learning object help? *Computers & Education*, 85, 203‑210. https://doi.org/10.1016/j.compedu.2015.02.017 |
| 000350337600013 | Wilson, T. L., & Hobolt, S. B. (2015). Allocating Responsibility in Multilevel Government Systems: Voter and Expert Attributions in the European Union*. Journal of Politics*, 77(1), 102‑113. https://doi.org/10.1086/678309 |
| 000353065700007 | Zhang, B., & Zhu, Z. (2015). A modified quasi-Newton diagonal update algorithm for total variation denoising problems and nonlinear monotone equations with applications in compressive sensing. *Numerical Linear Algebra with Applications*, 22(3), 500‑522. https://doi.org/10.1002/nla.1968 |
| 000353204900002 | Sciuto, F., Rosso, A., Sanfilippo, R., & Maniscalco, R. (2015). New faunistic data on the Pleistocene environmental evolution of the south-western edge of the Hyblean Plateau (SE Sicily). *Carnets De Geologie*, 15(4‑5), 41‑57. |
| 000357274600063 | Dobrovolskas, V., Kučinskas, A., Bonifacio, P., Caffau, E., Ludwig, H.-G., Steffen, M., & Spite, M. (2015). Three-dimensional hydrodynamical CO5BOLD model atmospheres of red giant stars - IV. Oxygen diagnostics in extremely metal-poor red giants with infrared OH lines. *Astronomy & Astrophysics*, 576, A128. https://doi.org/10.1051/0004-6361/201424885 |
| 000355321800013 | Jin, G., & Gaherty, J. B. (2015). Surface wave phase-velocity tomography based on multichannel cross-correlation. *Geophysical Journal International*, 201(3), 1383‑1398. https://doi.org/10.1093/gji/ggv079 |
| 000365808000014 | Pandey, A., Malek, V., Prabhakar, V., Kulkarni, Y. A., & Gaikwad, A. B. (2015). Nanoparticles: A Neurotoxicological Perspective. *Cns & Neurological Disorders-Drug Targets*, 14(10), 1317‑1327. https://doi.org/10.2174/1871527314666150821112411 |
| 000352854700010 | Nilson, F., Bonander, C., & Jonsson, A. (2015). Differences in Determinants Amongst Individuals Reporting Residential Fires in Sweden: Results from a Cross-Sectional Study. *Fire Technology*, 51(3), 615‑626. https://doi.org/10.1007/s10694-015-0459-0 |
| 000349266800011 | Garcia-Gomez, P., Hernandez-Quevedo, C., Jimenez-Rubio, D., & Oliva-Moreno, J. (2015). Inequity in long-term care use and unmet need: Two sides of the same coin. *Journal of Health Economics*, 39, 147‑158. https://doi.org/10.1016/j.jhealeco.2014.11.004 |
| 000356594900001 | Si, T., Zhang, K., Tang, J., Fang, M., Li, K., Zhuo, J., & Feng, Y. (2015). Efficacy and safety of flexibly dosed paliperidone palmitate in Chinese patients with acute schizophrenia: an open-label, single-arm, prospective, interventional study. *Neuropsychiatric Disease and Treatment*, 11, 1483‑1492. https://doi.org/10.2147/NDT.S81760 |
| 000353959400005 | Ando, K., Yorifuji, K., Ohnuma, S., Matthies, E., & Kanbara, A. (2015). Transmitting pro-environmental behaviours to the next generation: A comparison between Germany and Japan. *Asian Journal of Social Psychology*, 18(2), 134‑144. https://doi.org/10.1111/ajsp.12078 |
| 000346498800018 | Bischoff, A. L., Folsgaard, N. V., Vissing, N. H., Birch, S., Brix, S., & Bisgaard, H. (2015). Airway Mucosal Immune-suppression in Neonates of Mothers Receiving A(H1N1)pnd09 Vaccination During Pregnancy. *Pediatric Infectious Disease Journal*, 34(1), 84‑90. https://doi.org/10.1097/INF.0000000000000529 |
| 000362588800005 | Camejo, C. C., & Warnecke, G. (2015). The singular kernel coagulation equation with multifragmentation. *Mathematical Methods in the Applied Sciences*, 38(14), 2953‑2973. https://doi.org/10.1002/mma.3272 |
| 000347756900044 | Cuss, C. W., & Gueguen, C. (2015). Relationships between molecular weight and fluorescence properties for size-fractionated dissolved organic matter from fresh and aged sources. *Water Research*, 68, 487‑497. https://doi.org/10.1016/j.watres.2014.10.013 |
| 000350024900008 | Arashi, M., Bekker, A., Loots, M. T., & Roux, J. J. J. (2015). Integral Representation of Quaternion Elliptical Density and its Applications. *Communications in Statistics-Theory and Methods*, 44(4), 778‑789. https://doi.org/10.1080/03610926.2012.753089 |
| 000363704000011 | Gogebakan, T., & Eraslan, G. (2015). Single-dose toxicokinetics of permethrin in broiler chickens. *British Poultry Science*, 56(5), 605‑611. https://doi.org/10.1080/00071668.2015.1085957 |
| 000361400900022 | Song, X., Gu, H., Tang, L., Zhao, S., Zhang, X., Li, L., & Huang, J. (2015). Application of artificial bee colony algorithm on surface wave data. *Computers & Geosciences*, 83, 219‑230. https://doi.org/10.1016/j.cageo.2015.07.010 |
| 000347714700003 | Couceiro, M., Lehtonen, E., & Schölzel, K. (2015). A complete classification of equational classes of threshold functions included in clones. *RAIRO - Operations Research*, 49(1), 39‑66. https://doi.org/10.1051/ro/2014034 |
| 000352374400001 | Brancaccio, A., & Palacios, D. (2015). Chromatin signaling in muscle stem cells: interpreting the regenerative microenvironment. *Frontiers in Aging Neuroscience*, 7, 36. https://doi.org/10.3389/fnagi.2015.00036 |
| 000364362900035 | Li, Y., & Hardage, B. A. (2015). SV-P extraction and imaging for far-offset vertical seismic profile data. *Interpretation-a Journal of Subsurface Characterization*, 3(3), SW27‑SW35. https://doi.org/10.1190/INT-2015-0002.1 |
| 000348882900009 | Chen, C., Liu, C., Chen, C., Moyzis, R., Chen, W., & Dong, Q. (2015). Genetic variations in the serotoninergic system and environmental factors contribute to aggressive behavior in Chinese adolescents. *Physiology & Behavior*, 138, 62‑68. https://doi.org/10.1016/j.physbeh.2014.09.005 |
| 000345586900003 | Chong, H., Qiu, Z., Su, Y., Yang, L., & He, Y. (2015). Design of a highly potent HIV-1 fusion inhibitor targeting the gp41 pocket. *Aids*, 29(1), 13‑21. https://doi.org/10.1097/QAD.0000000000000498 |
| 000361977300090 | Atkinson, L. (2015). Locating the Politics in Political Consumption: A Conceptual Map of Four Types of Political Consumer Identities. *International Journal of Communication*, 9, 2047‑2066. |
| 000364777400031 | Rowland, L., Lobo-do-Vale, R. L., Christoffersen, B. O., Melem, E. A., Kruijt, B., Vasconcelos, S. S., … Meir, P. (2015). After more than a decade of soil moisture deficit, tropical rainforest trees maintain photosynthetic capacity, despite increased leaf respiration. *Global Change Biology*, 21(12), 4662‑4672. https://doi.org/10.1111/gcb.13035 |
| 000346267600010 | Krbalek, M., & Sleis, J. (2015). Vehicular headways on signalized intersections: theory, models, and reality. *Journal of Physics A-Mathematical and Theoretical*, 48(1), 015101. https://doi.org/10.1088/1751-8113/48/1/015101 |
| 000369908800022 | Zhou, J., Wang, J., Zeng, Y., Zhang, X., Hu, Q., Zheng, J., … Zhang, W.-M. (2015). Implication of epithelial-mesenchymal transition in IGF1R-induced resistance to EGFR-TKIs in advanced non-small cell lung cancer. *Oncotarget*, 6(42), 44332‑44345. https://doi.org/10.18632/oncotarget.6293 |
| 000344595900005 | Lotfalian, M., Ranjbar, M., Fazaelipoor, M. H., Schaffie, M., & Manafi, Z. (2015). Continuous Bioleaching of Chalcopyritic Concentrate at High Pulp Density. *Geomicrobiology Journal*, 32(1), 42‑49. https://doi.org/10.1080/01490451.2014.921256 |
| 000350285300006 | Schuster, L., & Majidi, N. (2015). Deportation Stigma and Re-migration. *Journal of Ethnic and Migration Studies*, 41(4), 635‑652. https://doi.org/10.1080/1369183X.2014.957174 |
| 000349637500005 | Naim, H. Y. (2015). Measles virus A pathogen, vaccine, and a vector. *Human Vaccines & Immunotherapeutics*, 11(1), 21‑26. https://doi.org/10.4161/hv.34298 |
| 000363471600015 | McGurk, R. C., Max, C. E., Medling, A. M., Shields, G. A., & Comerford, J. M. (2015). Spatially Resolved Imaging and Spectroscopy of Candidate Dual Active Galactic Nuclei. *Astrophysical Journal*, 811(1), 14. https://doi.org/10.1088/0004-637X/811/1/14 |
